# Exploring Methodological Issues in Mental Practice for Upper-Extremity Function Following Stroke-Related Paralysis: A Scoping Review

**DOI:** 10.3390/brainsci14030202

**Published:** 2024-02-22

**Authors:** Akira Nakashima, Ryohei Okamura, Takefumi Moriuchi, Kengo Fujiwara, Toshio Higashi, Kounosuke Tomori

**Affiliations:** 1 Graduate School of Biomedical Sciences, Nagasaki University, Nagasaki 852-8520, Japan; 2 Major of Occupational Therapy, Department of Rehabilitation, School of Health Science, Tokyo University of Technology, Tokyo 144-8535, Japan

**Keywords:** mental practice, stroke, upper-limb function, methodology

## Abstract

In this scoping review, we aimed to comprehensively clarify the methodology of Mental practice (MP) by systematically mapping studies documenting the application of MP to post-stroke paralytic upper-extremity function. Specifically, when is an MP intervention most commonly applied after stroke onset? What is the corresponding MP load (intervention time, number of intervention days, and intervention period)? What are the most common methods of Motor Imagery (MI) recall and MI tasks used during the application of MP? Is MP often used in conjunction with individual rehabilitation? What are the paralyzed side’s upper-limb and cognitive function levels at the start of an MP intervention? The research questions were identified according to PRISMA-ScR. The PubMed, Scopus, Medline, and Cochrane Library databases were used to screen articles published until 19 July 2022. In total, 694 English-language articles were identified, of which 61 were finally included. Most of the studies were conducted in the chronic phase after stroke onset, with limited interventions in the acute or subacute phase. The most common intervention time was ≤30 min and intervention frequency was 5 times/week in MP. An audio guide was most commonly used to recall MI during MP, and 50 studies examined the effects of MP in combination with individual rehabilitation. The Fugl-Meyer Assessment mean for the 38 studies, determined using the Fugl-Meyer Assessment, was 30.3 ± 11.5. Additional research with the aim of unifying the widely varying MP methodologies identified herein is warranted.

## 1. Introduction

Stroke is a typical target disease in rehabilitation. The factors that cause stroke patients to require support in their daily lives include the appearance of symptoms such as motor paralysis, sensory disturbances, and higher-brain dysfunction. Among these, motor paralysis significantly impacts daily life and quality of life, and improvement through rehabilitation is greatly required.

In this context, Mental Practice (MP) is an intervention that can be used to rehabilitate gait, balance, and upper-limb function after a stroke. MP is the continuous repetition of the presentation of Motor Imagery (MI) to improve performance on motor tasks, and its usefulness has been reported in systematic reviews on stroke patients [1,2]. Based on the results of many such studies, MP is also classified as Grade A in guidelines published by the American Heart Association [3].

However, it has been pointed out that there are no standardized intervention methods for the implementation of MP for gait, balance, or upper-limb function after stroke because of the wide variety of intervention parameters, such as MP intervention time, intervention frequency, and intervention duration [4,5]. Specifically, the time of MP implementation varies from 10 [6,7] to 60 min [8,9], the frequency of weekly intervention varies from two [10,11,12] to seven times a week [13,14], and the duration of an intervention varies from 3 [15] to 10 weeks [16,17]. Other factors such as the time from stroke onset to the start of the MP intervention, the status of physical function at the start of MP application, and the method of recalling motor imagery when performing MP (with audio guidance and action observation) differ among studies, and there is a lack of methodological consistency. In other words, the clinical use of MP to improve gait, balance, and upper-limb function after stroke is left to the subjective judgment of practitioners, and the development of intervention methods is necessary for future development.

This scoping review focuses on MP for paralytic upper-limb function, with the aim of understanding the current status of and identifying problems regarding more effective MP for paralytic upper-limb function and its further application in clinical practice. This scoping review systematically maps studies of MP for post-stroke paralytic upper-limb function and comprehensively clarifies the methodology of MP that has been used to date.

## 2. Materials and Methods

Our scoping review methodology was originally conceived by Arksey and O’Malley [18], developed in detail by Levac et al. [19], and implemented based on “Preferred Reporting Items for Systematic Reviews and Meta-Analyses Extension for Scoping Review (PRISMA-ScR)” as compiled by Triccol et al. [20]. We structured our protocol by applying a four-step process: identifying the research question, identifying the studies, selecting the studies, and extracting and analyzing the data.

Step 1: Identifying the research question

The purpose of this scoping review was to comprehensively clarify the methodology of MP to date by systematically mapping studies documenting the application of MP to post-stroke paralytic upper-extremity function. We used PCO to identify the research questions (Table 1). Specifically, (1) when is an MP intervention most commonly employed after stroke onset? (2) What is the MP load (intervention time, number of intervention days, and intervention period)? (3) What are the most common methods of MI recall and MI tasks used during MP? (4) Is MP often used in conjunction with individual rehabilitation? (5) What are the paralyzed side’s upper-limb and cognitive function levels at the start of an MP intervention?

Step 2: Identifying relevant studies

We searched for articles that included the words “stroke” and “mental practice (motor imagery training).” The databases used were PubMed, Scopus, Medline, and the Cochrane Library; the last search date was 19 July 2022. Free-text terms and Boolean operators (AND/OR) were applied when searching for titles or abstracts. No filters or limits were used. These keywords were chosen to encompass studies in which MP was applied to treat post-stroke paralytic upper-limb function. The search strategy used for each database is shown in Table 2.

“cerebrovascular disorder” OR stroke OR “Brain infarction” OR “Brain Stem Infarction” OR “Cerebral Infarction” OR Lacunar OR “Brain injury”

AND

“mental practice” OR “motor imagery training” OR “motor image”

Duplicate papers were removed after extracting papers from each database.

Step 3: Study selection

Papers meeting our criteria were selected from among English-language publications, and all study designs were included, encompassing those in which MP was applied to treat paralyzed upper-limb function after stroke. Five authors selected eligible articles using the Rayyan literature-screening software product (https://www.rayyan.ai/). For each article, the first author (AN) and two others (from among TM, KF, RO, or TH) independently read the title and abstract to exclude irrelevant papers and then read the full text, checking whether it met the eligibility criteria. In case of disagreement, all five authors reviewed the manuscript until 100% agreement was reached. Thereafter, AN and TM subsequently identified the levels of evidence and study designs of eligible articles using the American Journal of Occupational Therapy’s systematic review guidelines (https://research.aota.org/DocumentLibrary/AOTA_AJOT_systematic%20reviews%20instructions.pdf accessed on 13 November 2023).

Step 4: Data extraction and analysis

The following information was extracted from the eligible articles: author, year of publication, study design, country of study, age of participants with MP, type of stroke, timing of MP intervention, cognitive function at the start of the MP intervention, paralytic upper-limb function at the start of the MP intervention, duration of MP intervention, daily MP intervention time, length of MP intervention in days per week, how MI was performed during the MP intervention, and whether MP was combined with individual rehabilitation therapy.

## 3. Results

**Figure 1:**
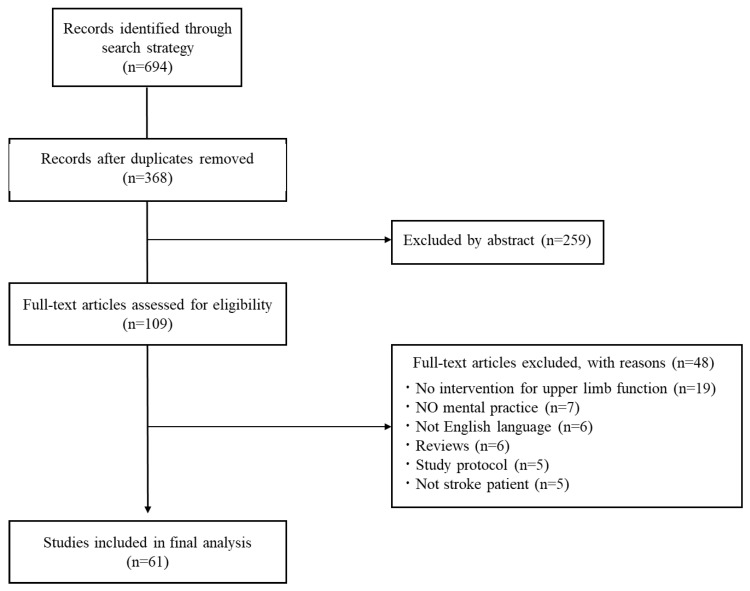
Flow diagram for inclusion and exclusion of studies. Figure format is derived from [71].

In total, 694 English-language articles were identified, of which 61 were selected for inclusion (Figure 1). The 61 articles selected are listed in Table 1. The study designs included 26 randomized controlled trials, 16 pre/post comparisons, 11 case series, four quasi-randomized controlled trials, four single case studies, and one crossover comparison study. Evaluation of each study using the American Journal of Occupational Therapy’s systematic review guidelines revealed qualitative problems regarding methodology for many studies (Table 3). The largest number of participants in each study was between 11 and 20 (18 studies), and 12 included more than 30 participants, of which the largest number was 121 [21]. The participants in each study were between 51 and 70 years old in the majority of the 48 studies. Only a single study included participants aged 70 or older [22].

(1) When is the most common time to start an MP intervention for post-stroke paralytic side upper-limb function after stroke?

In the study that started an MP intervention the earliest, the intervention was initiated 27.8 ± 19.2 days after stroke onset [23], and in the study that started an MP intervention the latest, the intervention was started 72.2 ± 20.3 months after onset [24]. Most of the studies were conducted in the chronic phase after stroke onset, and very few interventions were conducted in the acute or subacute phases. Table 3 shows the starting times of the MP interventions for all the studies.

(2) What is the MP load (intervention time, number of intervention days, and intervention period)?

The intervention times for MP varied across the studies: 13 studies had MP intervention times of 20 min or less, 26 studies had MP intervention times of 30 min or less, 13 studies had MP intervention times of 60 min or less, 1 study had MP intervention times of 60 min or longer, and 8 studies lacked information concerning MP intervention time. The study with the longest MP intervention time was that conducted by Butler et al. [25], with a value of 180 min. Regarding intervention frequency, 23 studies reported five weekly interventions, followed by 15 with three weekly interventions, 11 with two weekly interventions, and 7 with seven weekly interventions. There were no studies in which an MP intervention was performed once per week or six times a week. Five studies did not mention any MP. Next, regarding the duration of the MP interventions in each study, 4 weeks was the most common (18 studies), followed by 6 weeks (14 studies), 2 weeks, and 10 weeks (7 studies). The most common combination of intervention time per intervention, intervention frequency per week, and overall intervention period were 30 min per intervention and five times per week for 4 weeks (five studies) [26,27,28,29,30], followed by four papers with 45 min per intervention, five times per week, and 4 weeks [21,31,32,33].

**Table 3:** Levels of evidence and forms of intervention for the articles included in this scoping review.

No.	Author/Year	Paper Title	Evidence Level/ Study Design	Time Taken to Start Mental Practice Intervention after Stroke Onset	MP Combination	MP Intervention
1	Park et al. (2022) [24]	The effects of task-oriented mental practice on upper limb function and coordination in chronic stroke patients Randomized controlled trial design	2B/RCT	72.22 ± 20.37 months	Traditional rehabilitation	Time taken per intervention: 5 min Intervention frequency per week: 5/W Total intervention period: 3 weeks
2	Xiong et al. (2021) [26]	Motor imagery training reduces contralesional compensation in stroke patients with moderate to severe upper limb impairment	3B/One group, nonrandomized	112.08 ± 37.87 days	Traditional rehabilitation	Time taken per intervention: 30 min Intervention frequency per week: 5/W Total intervention period: 4 weeks
3	Qian Hu et al. (2021) [34]	Motor imagery-based brain-computer interface combined with multimodal feedback to promote upper limb motor function after stroke: A preliminary study	2B/RCT	7.9 ± 6.5 months	Traditional rehabilitation	Time taken per intervention: 30 min Intervention frequency per week: None Total intervention period: performed only once
4	Liepert et al. (2020) [35]	Effects of a single mental chronometry training session in subacute stroke patients—a randomized controlled trial	3B/One group, nonrandomized	2.1 ± 1.1 months	Traditional rehabilitation	Time taken per intervention: 30 min Intervention frequency per week: None Total intervention period: performed only once
5	Page et al. (2021) [36]	Multimodal Mental Practice Versus Repetitive Task Practice Only to Treat Chronic Stroke: A Randomized Controlled Pilot Study	2B/RCT	1.9 ± 2.5 months	Traditional rehabilitation	Time taken per intervention: 45 min Intervention frequency per week: 3/W Total intervention period: 10 weeks
6	Kang et al. (2021) [23]	The effects of additional electrical stimulation combined with repetitive transcranial magnetic stimulation and motor imagery on upper extremity motor recovery in the subacute period after stroke	2B/Tow group, nonrandomized	24.13 ± 12.4 days	Traditional rehabilitation	Time taken per intervention: 20 min Intervention frequency per week: 5/W Total intervention period: 2 weeks
7	Ji et al. (2021) [14]	Graded motor imagery training as a home exercise program for upper limb motor function in patients with chronic stroke A randomized controlled trial	2B/RCT	46.29 ± 40.96 months	Traditional rehabilitation	Time taken per intervention: 30 min Intervention frequency per week: 7/W Total intervention period: 8 weeks
8	Wang et al. (2020) [28]	Motor Imagery Training After Stroke Increases Slow-5 Oscillations and Functional Connectivity in the Ipsilesional Inferior Parietal Lobule.	2B/RCT	121.19 ± 37.33 days	Traditional rehabilitation	Time taken per intervention: 30 min Intervention frequency per week: 5/W Total intervention period: 4 weeks
9	Nam et al. (2019) [37]	Effects of adjuvant mental practice using inverse video of the unaffected upper limb in subacute stroke: a pilot randomized controlled study.	2B/RCT	67.4 ± 43.7 months	Traditional rehabilitation	Time taken per intervention: 20 min Intervention frequency per week: 5/W Total intervention period: 4 weeks
10	Kim et al. (2018) [6]	The effects of mental practice combined with modified constraint-induced therapy on corticospinal excitability, movement quality, function, and activities of daily living in persons with stroke.	2B/RCT	41 months (range: 8–120)	Traditional rehabilitation	Time taken per intervention: 10 min Intervention frequency per week: 5/W Total intervention period: 2 weeks
11	Chowdhury et al. (2018) [38]	Active Physical Practice Followed by Mental Practice Using BCI-Driven Hand Exoskeleton: A Pilot Trial for Clinical Effectiveness and Usability.	3B/One group, nonrandomized	7 ± 1.1 months	Traditional rehabilitation	Time taken per intervention: 30 min Intervention frequency per week: 2–3/WTotal intervention period: 6 weeks
12	Kawakami et al. (2018) [39]	Change in Reciprocal Inhibition of the Forearm with Motor Imagery among Patients with Chronic Stroke.	3B/One group, nonrandomized	30.5 months (range 9~180)	Traditional rehabilitation	Time taken per intervention: 45 min Intervention frequency per week: 5/W Total intervention period: 10 days
13	Fang et al. (2018) [31]	Motor imagery training induces changes in brain neural networks in stroke patient	2B/RCT	1.8 ± 0.7 months	Traditional rehabilitation	Time taken per intervention: 45 min Intervention frequency per week: 5/W Total intervention period: 4 weeks
14	Azad et al. (2018) [40]	Effect of motor imagery training with sensory feedback on sensory-motor function of the upper extremity in patients with chronic stroke	2B/Tow group, nonrandomized	None	Traditional rehabilitation	Time taken per intervention: None Intervention frequency per week: None Total intervention period: None
15	Park et al. (2017) [27]	Effects of mental practice combined with electromyogram-triggered electrical stimulation for upper extremity function in stroke patients	2B/Tow group, nonrandomized	None	Traditional rehabilitation	Time taken per intervention: 30 min Intervention frequency per week: 5/W Total intervention period: 4 weeks
16	Iso at al. (2016) [41]	Effect of mental practice using inverse video of the unaffected upper limb in a subject with chronic hemiparesis after stroke	4/Case study	5 years	Traditional rehabilitation	Time taken per intervention: 30 min Intervention frequency per week: 5/W Total intervention period: 6 weeks
17	Park et al. (2016) [42]	Influence of mental practice on upper limb muscle activity and activities of daily living in chronic stroke patients	4/Case series	39.5 ± 3.5 months	Traditional rehabilitation	Time taken per intervention: 30 min Intervention frequency per week: 5/W Total intervention period: 2 weeks
18	Park et al. (2016) [43]	The effects of game-based virtual reality movement therapy plus mental practice on upper extremity function in chronic stroke patients with hemiparesis: A randomized controlled trial	2B/RCT	More than 6 months	VR rehabilitation	Time taken per intervention: 5 min Intervention frequency per week: 5/W Total intervention period: 2 weeks
19	Oh et al. (2016) [44]	Effects of Adjuvant Mental Practice on Affected Upper Limb Function Following a Stroke: Results of Three-Dimensional Motion Analysis, Fugl-Meyer Assessment of the Upper Extremity and Motor Activity Log	2B/Tow group, nonrandomized	128.1 ± 26.05 days	Traditional rehabilitation	Time taken per intervention: 20 min Intervention frequency per week: 3/W Total intervention period: 3 weeks
20	Page et al. (2016) [45]	Retention of the spacing effect with mental practice in hemiparetic stroke.	2B/RCT	1041.5 ± 999.8 days	Traditional rehabilitation	Time taken per intervention: 60 min Intervention frequency per week: 3/W Total intervention period: 10 weeks
21	Cha et al. (2015) [46]	Effects of mental practice with action observation training on occupational performance after stroke.	4/Case series	25.3 ± 14 months	Traditional rehabilitation	Time taken per intervention: 4 min Intervention frequency per week: None Total intervention period: 20 times
22	Morone et al. (2015) [47]	Proof of principle of a brain-computer interface approach to support poststroke arm rehabilitation in hospitalized patients: design, acceptability, and usability.	3B/One group, nonrandomized	24.3 ± 21.1 days	MP only	Time taken per intervention: 30 min Intervention frequency per week: 3/W Total intervention period: 4 weeks
23	Page et al. (2015) [13]	Mental Practice–Triggered Electrical Stimulation in Chronic, Moderate, Upper-Extremity Hemiparesis After Stroke	4/Case series	56.5 ± 42.2 months	Electrical stimulation	Time taken per intervention: 60 min Intervention frequency per week: 7/W Total intervention period: 8 weeks
24	Park et al. (2015) [7]	Effects of mental practice on stroke patients’ upper extremity function and daily activity performance	2B/RCT	18 ± 11.7 months	Traditional rehabilitation	Time taken per intervention: 10 min Intervention frequency per week: 5/W Total intervention period: 2 weeks
25	Park et al. (2015) [48]	The effects of modified constraint-induced therapy combined with mental practice on patients with chronic stroke	2B/RCT	15.9 ± 5.8 months	Traditional rehabilitation	Time taken per intervention: 30 min Intervention frequency per week: 5/W Total intervention period: 6 weeks
26	Bajaj et al. (2015) [49]	Functional organization and restoration of the brain motor-execution network after stroke and rehabilitation	2B/Tow group, nonrandomized	10.1 ± 13.3 months	Traditional rehabilitation or MP only	Time taken per intervention: 240 min Intervention frequency per week: None Total intervention period: 3 weeks (3600 min)
27	Kim et al. (2015) [50]	Motor imagery training improves upper extremity performance in stroke patients	2B/RCT	8.1 months	Traditional rehabilitation	Time taken per intervention: 30 min Intervention frequency per week: 3/W Total intervention period: 4 weeks
28	Hua et al. (2014) [32]	Changes in brain activation in stroke patients after mental practice and physical exercise a functional MRI study	2B/Tow group, nonrandomized	1.61 ± 0.8 months	Traditional rehabilitation	Time taken per intervention: 45 min Intervention frequency per week: 5/W Total intervention period: 4 weeks
29	Oliveira et al. (2014) [51]	Mental practice and mirror therapy associated with conventional physical therapy training on the hemiparetic upper limb in poststroke rehabilitation: a preliminary study.	3B/One group, nonrandomized	4.14 ± 1.9 months	Traditional rehabilitation	Time taken per intervention: 25 min Intervention frequency per week: 2/W Total intervention period: 8 weeks
30	de Assis et al. (2014) [52]	An augmented reality system for upper-limb post-stroke motor rehabilitation: a feasibility study.	3B/One group, nonrandomized	None	None	Time taken per intervention: 60 min Intervention frequency per week: 1~2/W Total intervention period: 4 weeks
31	Ji et al. (2014) [53]	Effects of Mental Practice in Conjunction with Repetitive Transcranial Magnetic Stimulation on the Upper Limbs of Sub-acute Stroke Patients	2B/RCT	7.81 ± 2.4 months	Traditional rehabilitation	Time taken per intervention: 15 min Intervention frequency per week: 5/W Total intervention period: 6 weeks
32	Liu et al. (2014) [33]	Mental practice combined with physical practice to enhance hand recovery in stroke patients.	2B/Tow group, nonrandomized	1.83 ± 0.6 months	Traditional rehabilitation	Time taken per intervention: 45 min Intervention frequency per week: 5/W Total intervention period: 4 weeks
33	Sun et al. (2013) [29]	Cortical reorganization after motor imagery training in chronic stroke patients with severe motor impairment: a longitudinal fMRI study.	2B/RCT	132.1 ± 27.3 days	Traditional rehabilitation	Time taken per intervention: 30 min Intervention frequency per week: 5/W Total intervention period: 4 weeks
34	Clarissa et al. (2013) [54]	The addition of functional task-oriented mental practice to conventional physical therapy improves motor skills in daily functions after stroke.	4/Case series	13 ± 6.5 months	Traditional rehabilitation	Time taken per intervention: 30 min Intervention frequency per week: 3/W Total intervention period: 4 weeks
35	Mihara et al. (2013) [55]	Near-infrared spectroscopy-mediated neurofeedback enhances efficacy of motor imagery-based training in poststroke victims: a pilot study.	2B/RCT	135 ± 38.2 days	Traditional rehabilitation	Time taken per intervention: 30 min Intervention frequency per week: 3/W Total intervention period: 2 weeks
36	Timmermans et al.(2013) [56]	Effect of mental practice on the improvement of function and daily activity performance of the upper extremity in patients with subacute stroke: a randomized clinical trial.	2B/RCT	36.1 ± 27.4 days	MP only	Time taken per intervention: 30 min Intervention frequency per week: 7/W Total intervention period: 6 weeks
37	Nilsen et al. (2012) [12]	Effect of imagery perspective on occupational performance after stroke: a randomized controlled trial.	2B/RCT	43.2 ± 15.4 months	Traditional rehabilitation	Time taken per intervention: 18 min Intervention frequency per week: 2/W Total intervention period: 6 weeks
38	Braun et al. (2012) [22]	A multicenter randomized controlled trial to compare subacute ‘treatment as usual’ with and without mental practice among persons with stroke in Dutch nursing homes.	2B/RCT	6.1 ± 2.7 months	Traditional rehabilitation	Time taken per intervention: None Intervention frequency per week: None Total intervention period: 6 weeks
39	Trobia et al. (2011) [57]	Combined use of music and virtual reality to support mental practice in stroke rehabilitation	4/Case series	24 months	MP only	Time taken per intervention: None Intervention frequency per week: 7/W Total intervention period: 4 weeks
40	Page et al. (2011) [58]	Retention of motor changes in chronic stroke survivors who were administered mental practice.	2B/RCT	58.7 months (range 13–129)	Traditional rehabilitation	Time taken per intervention: 30 min Intervention frequency per week: 5/W Total intervention period: 10 weeks
41	Page et al. (2011) [59]	Longer versus shorter mental practice sessions for affected upper extremity movement after stroke: a randomized controlled trial.	2B/RCT	36 months	Traditional rehabilitation	Time taken per intervention: 20 or 40 or 60 min Intervention frequency per week: 3/W Total intervention period: 10 weeks
42	Ietswaart et al. (2011) [21]	Mental practice with motor imagery in stroke recovery: randomized controlled trial of efficacy	1B/RCT	82 ± 55 days	MP only	Time taken per intervention: 45 min Intervention frequency per week: 5/W Total intervention period: 4 weeks
43	Wu et al. (2011) [60]	Improved function after combined physical and mental practice after stroke: a case of hemiparesis and apraxia.	4/Case study	7 months	Traditional rehabilitation	Time taken per intervention: 30 min Intervention frequency per week: 5/W Total intervention period: 6 weeks
44	Céline et al. (2010) [61]	Determining specificity of motor imagery training for upper limb improvement in chronic stroke patients: a training protocol and pilot results.	3B/One group, nonrandomized	16.5 ± 7.3	MP only	Time taken per intervention: 15 min Intervention frequency per week: 4/W Total intervention period: 3 weeks
45	Riccio et al.(2010) [15]	Mental practice is effective in upper limb recovery after stroke: a randomized single-blind cross-over study.	2B/RCT	7.33 ± 2.38 week	Traditional rehabilitation	Time taken per intervention: 60 min Intervention frequency per week: 5/W Total intervention period: 3 weeks
46	Gaggioli et al. (2009) [62]	Computer-guided mental practice in neurorehabilitation.	3B/One group, nonrandomized	31 ± 25.3 months	Traditional rehabilitation	Time taken per intervention: 30 min Intervention frequency per week: 2/W Total intervention period: 8 weeks
47	Prasad et al. (2009) [63]	Using Motor Imagery Based Brain-Computer Interface for Post-stroke Rehabilitation	4/Case series	28 ± 15.4 months	Traditional rehabilitation	Time taken per intervention: None Intervention frequency per week: 2/W Total intervention period: 6 weeks
48	Page et al. (2009) [16]	Cortical plasticity following motor skill learning during mental practice in stroke.	3B/One group, nonrandomized	36.7 ± 34 months	Traditional rehabilitation	Time taken per intervention: 30 min Intervention frequency per week: 5/W Total intervention period: 10 weeks
49	Page et al. (2009) [17]	Modified constraint-induced therapy combined with mental practice: thinking through better motor outcomes.	2B/RCT	28.5 months	Traditional rehabilitation	Time taken per intervention: 30 min Intervention frequency per week: 3/W Total intervention period: 10 weeks
50	Simmons et al. (2008) [64]	Motor imagery to enhance recovery after subcortical stroke: who might benefit, daily dose, and potential effects.	3B/One group, nonrandomized	8.71 ± 10.4 months	MP only	Time taken per intervention: 60 min Intervention frequency per week: 5/W Total intervention period: 2 weeks
51	Hewett et al. (2007) [10]	Reaching kinematics to measure motor changes after mental practice in stroke.	4/Case series	51.2 months	Traditional rehabilitation	Time taken per intervention: 30 min Intervention frequency per week: 2/W Total intervention period: 6 weeks
52	Page et al. (2007) [11]	Mental practice as a gateway to modified constraint-induced movement therapy: a promising combination to improve function.	4/Case series	32 ± 22 months	Traditional Rehabilitation	Time taken per intervention: None Intervention frequency per week: 2/W Total intervention period: 6 weeks
53	Page et al. (2007) [65]	Mental practice in chronic stroke: results of a randomized, placebo-controlled trial.	1B/RCT	38.8 ± 25.8 months	Traditional rehabilitation	Time taken per intervention: 30 min Intervention frequency per week: 2/W Total intervention period: 6 weeks
54	Müller et al. (2007) [30]	Mental practice improves hand function after hemiparetic stroke.	2B/RCT	28.7 ± 21.2 months	Traditional rehabilitation	Time taken per intervention: 30 min Intervention frequency per week: 5/W Total intervention period: 4 weeks
55	Butler et al. (2006) [25]	Mental practice with motor imagery: evidence for motor recovery and cortical reorganization after stroke.	4/Case series	9.2 ± 6.7 months	Traditional rehabilitation	Time taken per intervention: 180 min Intervention frequency per week: 7/W Total intervention period: 2 weeks
56	Gaggioli et al. (2005) [66]	A strategy for computer-assisted mental practice in stroke rehabilitation.	4/Case study	13 months	Traditional rehabilitation	Time taken per intervention: 30 min Intervention frequency per week: 3/W Total intervention period: 12 weeks
57	Page et al. (2005) [67]	Effects of mental practice on affected limb use and function in chronic stroke.	2B/RCT	23.8 months	Traditional rehabilitation	Time taken per intervention: 30 min Intervention frequency per week: 2/W Total intervention period: 6 weeks
58	Dijkerman et al. (2004) [68]	Does motor imagery training improve hand function in chronic stroke patients? A pilot study.	2B/RCT	2 ± 0.8 months	MP only	Time taken per intervention: None Intervention frequency per week: 7/W Total intervention period: 4 weeks
59	Stevens et al. (2003) [8]	Using motor imagery in the rehabilitation of hemiparesis	4/Case series	44 ± 42.4 months	MP only	Time taken per intervention: 60 min Intervention frequency per week: 3/W Total intervention period: 4 weeks
60	Crosbie et al. (2003) [69]	The adjunctive role of mental practice in the rehabilitation of the upper limb after hemiplegic stroke: a pilot study.	4/Case series	39.4 ± 49.6 days	Traditional rehabilitation	Time taken per intervention: 45 min Intervention frequency per week: 5/W Total intervention period: 2 weeks
61	Page et al. (2001) [70]	Mental practice combined with physical practice for upper-limb motor deficit in subacute stroke.	4/Case study	5 months	Traditional rehabilitation	Time taken per intervention: 10 min Intervention frequency per week: 2/W Total intervention period: 6 weeks

MP, mental practice; RCT, randomized controlled trial; None, no detailed description provided.

(3) What are the most common methods of MI recall and MI tasks used during an MP intervention?

The most common methods of recalling MI during an MP intervention consisted of using an audio guide to prompt MI while giving verbal instructions (22 studies), using MI alone (18 studies), and using MI combined with action observation (7 studies).

Other methods included using BCI, reported in five papers [34,38,39,47,63], and VR, reported in three papers [57,62,66].

The most common MI tasks used in MP were daily activities such as “drinking water from a glass” and “buttoning a shirt” (28 papers). Twenty-two studies used joint movements such as hand flexion and wrist dorsiflexion. Seven studies used both daily activities and joint exercises.

(4) Is MP often used in conjunction with individual rehabilitation?

A total of 49 studies examined the effects of MP in combination with individual rehabilitation, and 8 studies used MP alone (Table 3).

(5) What are the upper-limb and cognitive function levels on the paralyzed side at the start of an MP intervention?

In this scoping review, Fugl-Meyer Assessment (FMA), Action Research Arm Test (ARAT), Motor Activity Log (MAL), and Box and Block Test (BBT) data were extracted from each article to confirm the status of paralyzed-side upper-limb function at the start of an MP intervention. As a result, FMA, ARAT, MAL, and BBT were implemented in 38, 21, 9, and 6 studies. The FMA mean for the 38 studies using FMA was 30.3 ± 11.5.

Twelve studies measured cognitive function at the start of MP using the Mini-Mental State Examination (MMSE). Nineteen studies used MMSE > 24 or 25 or 27 as the inclusion criterion. Eleven studies used a Modified Mini-Mental State Examination score of > 69 or 70 as the inclusion criterion. Nineteen studies did not test cognitive function. Among the 12 studies that measured cognitive function using the MMSE at the beginning of the MP intervention, the minimum MMSE score was 25 ± 2 points [23].

## 4. Discussion

This scoping review aimed to systematically map studies in which an MP intervention was performed for treating post-stroke paralytic lateral upper-extremity function to provide a comprehensive picture of the MP methodologies used to date. When assessing these studies, our investigation considered the following: (1) When is the most common time to carry out an MP intervention after stroke onset? (2) What is the MP load (intervention time, number of intervention days, and intervention period)? (3) What are the most common methods of MI recall and MI tasks used during an MP intervention? (4) Is MP often used in conjunction with individual rehabilitation? (5) What are the upper-limb and cognitive function levels on the paralyzed side at the start of an MP intervention?

(1) When is an MP intervention most frequently employed after stroke onset?

Most studies were conducted in the chronic phase after stroke onset, and this scoping review suggests that MP is an effective intervention strategy for treating upper-limb function on the paralyzed side 3 months after stroke onset. However, very few studies have examined the effect of intervention in the acute phase of a stroke. The need for such an intervention can be inferred from how cognitive aspects have a significant impact on how an MP intervention is conducted and from the fact that the participant is unable to perform adequate MI during an MP intervention in the acute phase of stroke onset because of impaired consciousness. Further, from the viewpoint of research design, it is difficult to derive the effects of specific approaches for participants in the acute and subacute phases of stroke because there are many factors (cerebral edema, diaschisis, and improvement of penumbra) [72] that may improve physical function, and researchers are reluctant to publish negative data. However, studies concerning the acute and subacute phases of stroke onset are essential to determine the appropriate time to start an MP intervention, and future research should focus on the effectiveness of MP in the acute and subacute phases.

(2) What is the MP load (intervention time, number of intervention days, and intervention period)?

In all the studies, there were no clear criteria for the MP load, and the intervention time, days of intervention, and duration of intervention varied in a wide variety of situations. The only study that investigated MP loading was Page et al.’s study on the MP intervention period [59]. In addition, in recent years, when considering MP load, it has become clear that MI can cause muscle and mental fatigue with sustained repetition, which can also affect performance improvement [73,74,75,76,77]. Against this backdrop, systematic reviews on MP have pointed out the importance of formulating interventions that account for fatigue associated with sustained repetition of MI [78]. In the future, it will be important to cooperate with basic researchers to establish standardized intervention criteria for MP and investigate what level of load is most effective from a neurophysiological perspective.

(3) What are the most common methods of MI recall and MI tasks used during an MP intervention?

The most common method of conducting an MP intervention was the use of an audio guide to facilitate MI with verbal instructions, and it is important to know how MI can be performed to maximize the effectiveness of MP [79]. Several recent studies have begun to use VR and BCI to enhance MI clarity [34,38,39,47,57,62,63,66], and we believe that it will be important to apply MI clarifying techniques to optimize MP efficacy in the future. One treatment method whose effectiveness is being enhanced by this VR technology is mirror therapy (MT). MT is a treatment modality that induces cortical reorganization and promotes plastic changes in the brain without requiring movement of the affected limb [80]. Systematic reviews have also shown its effectiveness [81]. Additionally, the VR-based mirror therapy system (VRMT), which applies the concept of MT, is expected to be a more effective treatment method compared with conventional MT [82,83]. Systematic reviews have also reported that VRMT shows effectiveness when combined with traditional rehabilitation [84].

The most common tasks used during an MP intervention were tasks involving daily activities such as “drinking water from a glass” and “buttoning a shirt”. This may be because of the combination of task-oriented training, the ease of generalization to daily activities, and the use of familiar activities to ensure MI clarity. It is possible that employing an MP intervention for familiar or everyday activities may help to facilitate the process of activating the mentoring system [85].

(4) Is MP often used in conjunction with individual rehabilitation?

Motor imagery is defined as the “mental simulation” or “mental rehearsal” of movement without any actual body movement [86,87]. In fact, combining mental and physical training is effective in that the motor imagery practice can be useful for improving performance in rehabilitation programs [88,89,90]. Therefore, the effectiveness of MP is considered to be maximal when combined with individualized rehabilitation. In this context, the results of a study concerning two groups that performed 45 min of physical exercise and 15 min each of action observation, physical exercise, and MP activities with respect to the paralyzed side of the upper-limb showed that the group that combined action observation, physical exercise, and MP techniques exhibited improvements in upper-limb function on the paralyzed side [36]. These findings suggest that it may be necessary to not only combine these exercises in the future but also consider the order of the exercises and the allocation of time for each exercise within the overall practice period.

(5) What are the upper-limb and cognitive function levels on the paralyzed side at the start of MP intervention?

Depending on the FMA score, MP interventions tend to be applied to participants with mild-to-moderate paralysis and, according to MMSE, relatively preserved cognitive function. There are no indication criteria for MP concerning paralytic upper-limb function or cognitive status. For example, in CI therapy, the criteria for indication include the ability to perform a 10° extension of the MP and IP joints and a 20° dorsiflexion of the wrist in the paralyzed upper extremity and an MMSE score of 24 or higher in cognitive function [91]. In the case of MP, it is important to perform clear MI tasks to realize their effects fully, and the participant must understand the practitioner’s explanations. From this point of view, participants with relatively preserved cognitive functions are likely to benefit from MP. However, it is important to combine action observation and VR to ensure motor imagery ability and to prepare the environment so that even participants with diminished cognitive function can benefit from MP. Further accumulation of research data is needed to accumulate studies on people with severe paralytic upper-limb dysfunction and cognitive decline to investigate the extent to which people with paralytic upper-limb function and cognitive function can benefit from MP.

One limitation of this study was that it was a scoping review, so we did not evaluate the advantages and disadvantages offered by MP in each study. For this reason, it was not possible to describe the effectiveness of MP in rehabilitation interventions. In addition, although five experienced occupational therapists reviewed each study, we cannot deny the possibility that another occupational therapist or a different team of occupational therapists would have had a different opinion. Furthermore, many of the studies collected in this study were of low quality overall; therefore, the results of this study should be interpreted with caution. 

## 5. Clinical Implications

This scoping review aimed to systematically map studies in which MP was performed to adress post-stroke paralytic lateral upper extremity function to provide a comprehensive picture of the MP methodologies used to date. MP is used in the field of sports and rehabilitation to improve the performance of motor tasks through the continuously repeated presentation of motor imagery. The vividness with which a subject can recall images in motor imagery tasks is important for effective MP. In addition, it has become clear in recent years that fatigue occurs with the sustained repetition of motor images, and it may be necessary to pay attention to the load in order to implement effective MP. Currently, a wide variety of methodologies for MP exist for post-stroke paralytic upper-extremity function. In the future, it will be necessary to establish effective MP methodologies through higher-quality research.

## 6. Conclusions

In this study, we comprehensively reviewed the MP methodologies used to date for the rehabilitation of paralyzed upper-extremity function. We found that the duration of MP interventions varied widely and that many studies differed in their methods of MI recall. In the future, accumulating more data accumulated via studies performed in cooperation with basic and clinical researchers will be important to unify the widely varying MP methodologies identified in this study.

## Figures and Tables

**Table 1:** The process of identifying the research questions and inclusion criteria.

Identifying the Research Questions	
Participants	Adult stroke patient
Concept	MP (time taken to start the intervention; MP load; MI recall and MI tasks performed during MP; association with individual rehabilitation; function level for the use of MP)
Context	Acute to chronic; Japan; Abroad
**Inclusion Criteria**	
· A study on MP for post-stroke paralytic lateral upper-limb function (Including all study types) · English Papers	

**Table 2:** Full search strategies employed for each database.

Database	Search Strategy
PubMed	(“cerebrovascular disorder”[Title/Abstract] OR (“stroke”[MeSH Terms] OR “stroke”[Title/Abstract] OR “strokes”[Title/Abstract] OR “stroke s”[Title/Abstract]) OR “Brain infarction”[Title/Abstract] OR “Brain Stem Infarctions”[Title/Abstract] OR “Cerebral Infarction”[Title/Abstract] OR (“lacunar”[Title/Abstract] OR “lacunars”[Title/Abstract]) OR “Brain injury”[Title/Abstract]) AND (“mental practice”[Title/Abstract] OR “motor imagery training”[Title/Abstract] OR “motor image”[Title/Abstract])
Scopus	(TITLE-ABS-KEY (“cerebrovascular disorder” OR stroke OR “brain infarction” OR “brain stem infarctions” OR “cerebral infarction” OR lacunar OR “brain injury”)) AND (TITLE-ABS-KEY (“mental practice” OR “motor imagery training” OR “motor image”))
Medline	title(“cerebrovascular disorder” OR stroke OR “Brain infarction” OR “Brain Stem Infarctions” OR “Cerebral Infarction” OR Lacunar OR “Brain injury”) AND title(“mental practice” OR “motor imagery training” OR “motor image”) abstract(“cerebrovascular disorder” OR stroke OR “Brain infarction” OR “Brain Stem Infarctions” OR “Cerebral Infarction” OR Lacunar OR “Brain injury”) AND abstract(“mental practice” OR “motor imagery training” OR “motor image”)
Cochrane Library	Cochrane Reviews matching “cerebrovascular disorder” OR stroke OR “Brain infarction” OR “Brain Stem Infarctions” OR “Cerebral Infarction” OR Lacunar OR “Brain injury” in Title Abstract Keyword AND “mental practice” OR “motor imagery training” OR “motor image” in Title Abstract Keyword

## Data Availability

The datasets generated and/or analyzed during the current study are available from the corresponding author upon reasonable request.
